# Effect of rAd5-Vector HIV-1 Preventive Vaccines on HIV-1 Acquisition: A Participant-Level Meta-Analysis of Randomized Trials

**DOI:** 10.1371/journal.pone.0136626

**Published:** 2015-09-02

**Authors:** Yunda Huang, Dean Follmann, Martha Nason, Lily Zhang, Ying Huang, Devan V. Mehrotra, Zoe Moodie, Barbara Metch, Holly Janes, Michael C. Keefer, Gavin Churchyard, Merlin L. Robb, Patricia E. Fast, Ann Duerr, M. Juliana McElrath, Lawrence Corey, John R. Mascola, Barney S. Graham, Magdalena E. Sobieszczyk, James G. Kublin, Michael Robertson, Scott M. Hammer, Glenda E. Gray, Susan P. Buchbinder, Peter B. Gilbert

**Affiliations:** 1 Vaccine and Infectious Disease Division, Fred Hutchinson Cancer Research Center, Seattle, Washington, United States of America; 2 National Institute of Allergy and Infectious Diseases and Biostatistics Research Branch, National Institutes of Health, Bethesda, MD, United States of America; 3 Division of Clinical Research, National Institute of Allergy and Infectious Diseases, National Institutes of Health, Bethesda, MD, United States of America; 4 Merck Research Laboratories, North Wales, PA, United States of America; 5 Infectious Disease Division, University of Rochester School of Medicine and Dentistry, Rochester, NY, United States of America; 6 Aurum Institute for Health Research, Johannesburg, South Africa; 7 HJF HIV Program, US Military HIV Research Program, Bethesda, MD, United States of America; 8 Research and Development, International AIDS Vaccine Initiative, New York, New York, United States of America; 9 Vaccine Research Center, National Institute of Allergy and Infectious Diseases, Bethesda, MD, United States of America; 10 Viral Pathogenesis Laboratory, National Institute of Allergy and Infectious Diseases, Bethesda, MD, United States of America; 11 Division of Infectious Diseases, Department of Medicine, Columbia University, New York, New York, United States of America; 12 Infectious Disease Clinical Research, Merck, Philadelphia, Pennsylvania, United States of America; 13 University of the Witwatersrand, Johannesburg, South Africa; 14 Bridge HIV, San Francisco Department of Public Health, San Francisco, CA, United States of America; French National Centre for Scientific Research, FRANCE

## Abstract

**Background:**

Three phase 2b, double-blind, placebo-controlled, randomized efficacy trials have tested recombinant Adenovirus serotype-5 (rAd5)-vector preventive HIV-1 vaccines: MRKAd5 HIV-1 *gag/pol/nef* in Step and Phambili, and DNA/rAd5 HIV-1 *env/gag/pol* in HVTN505. Due to efficacy futility observed at the first interim analysis in Step and HVTN505, participants of all three studies were unblinded to their vaccination assignments during the study but continued follow–up. Rigorous meta-analysis can provide crucial information to advise the future utility of rAd5-vector vaccines.

**Methods:**

We included participant-level data from all three efficacy trials, and three Phase 1–2 trials evaluating the HVTN505 vaccine regimen. We predefined two co-primary analysis cohorts for assessing the vaccine effect on HIV-1 acquisition. The modified-intention-to-treat (MITT) cohort included all randomly assigned participants HIV-1 uninfected at study entry, who received at least the first vaccine/placebo, and the Ad5 cohort included MITT participants who received at least one dose of rAd5-HIV vaccine or rAd5-placebo. Multivariable Cox regression models were used to estimate hazard ratios (HRs) of HIV-1 infection (vaccine vs. placebo) and evaluate HR variation across vaccine regimens, time since vaccination, and subgroups using interaction tests.

**Findings:**

Results are similar for the MITT and Ad5 cohorts; we summarize MITT cohort results. Pooled across the efficacy trials, over all follow-up time 403 (n = 224 vaccine; n = 179 placebo) of 6266 MITT participants acquired HIV-1, with a non-significantly higher incidence in vaccine recipients (HR 1.21, 95% CI 0.99–1.48, P = 0.06). The HRs significantly differed by vaccine regimen (interaction *P* = 0.03; MRKAd5 HR 1.41, 95% CI 1.11–1.78, *P* = 0.005 vs. DNA/rAd5 HR 0.88, 95% CI 0.61–1.26, *P* = 0.48). Results were similar when including the Phase 1–2 trials. Exploratory analyses based on the efficacy trials supported that the MRKAd5 vaccine-increased risk was concentrated in Ad5-positive or uncircumcised men early in follow-up, and in Ad5-negative or circumcised men later. Overall, MRKAd5 vaccine-increased risk was evident across subgroups except in circumcised Ad5-negative men (HR 0.97, 95% CI 0.58−1.63, *P* = 0.91); there was little evidence that the DNA/rAd5 vaccine, that was tested in this subgroup, increased risk (HR 0.88, 95% CI 0.61–1.26, *P* = 0.48). When restricting the analysis of Step and Phambili to follow-up time before unblinding, 114 (n = 65 vaccine; n = 49 placebo) of 3770 MITT participants acquired HIV-1, with a non-significantly higher incidence in MRKAd5 vaccine recipients (HR 1.30, 95% CI 0.89–1.14, P = 0.18).

**Interpretation and Significance:**

The data support increased risk of HIV-1 infection by MRKAd5 over all follow-up time, but do not support increased risk of HIV-1 infection by DNA/rAd5. This study provides a rationale for including monitoring plans enabling detection of increased susceptibility to infection in HIV-1 at-risk populations.

## Introduction

**Fig 1:**
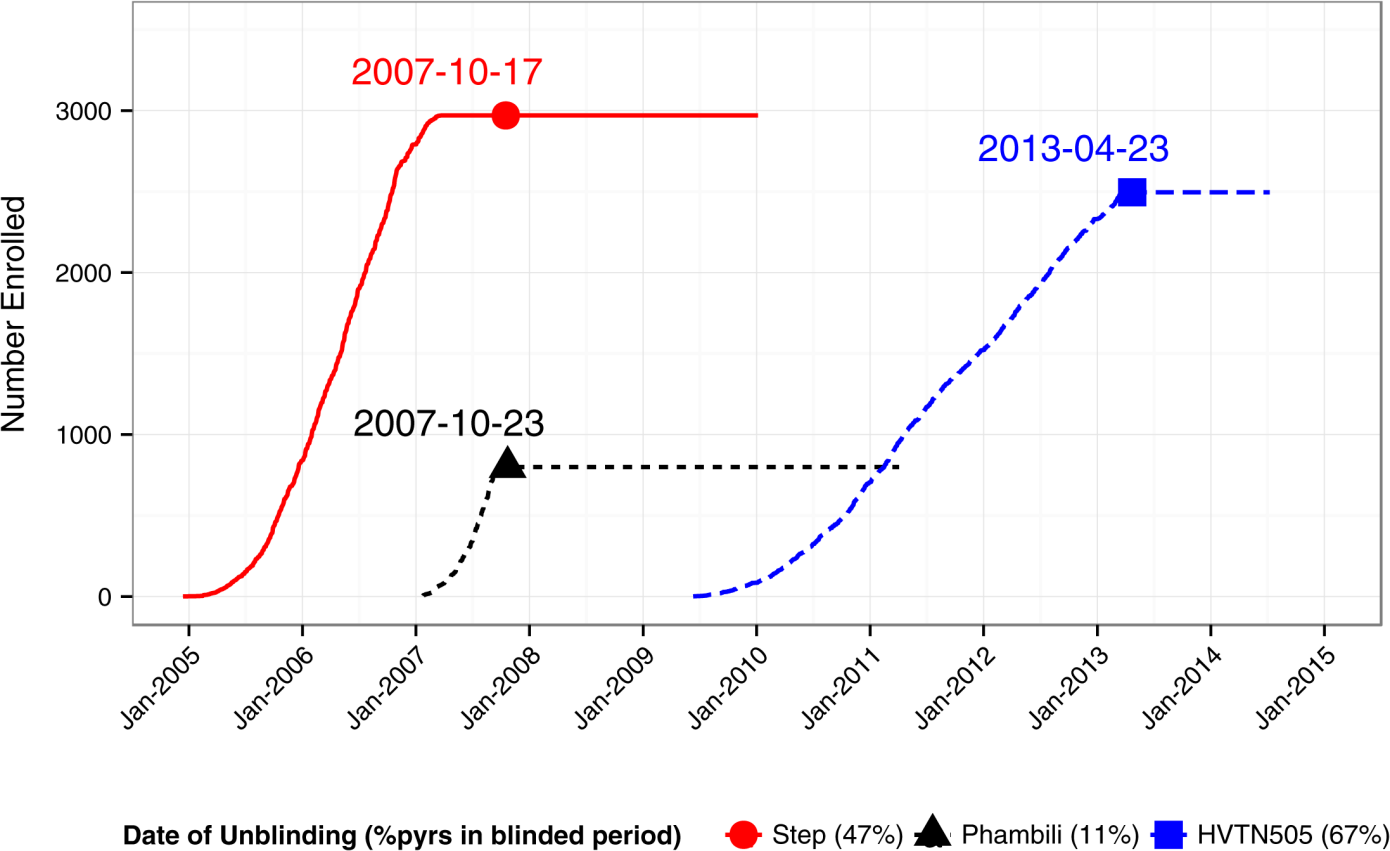
Enrollment and Follow-up in Step, Phambili and HVTN505. Indicated are dates of unblinding when data were frozen for pre-unblinding analyses, and the percent of person-years (% pyrs) occurring during the blinded period. HVTN505 is still in active follow-up; HVTN505 data included in the meta-analysis are as of July 9, 2014.

Credited to their immunological and manufacturing properties, replication-defective adenovirus (Ad) vectors have been one of the most explored delivery vehicles of vaccines for cancer and a variety of pathogens, including HIV-1, *Plasmodium falciparum*, *Mycobacterium tuberculosis*, hepatitis C, Ebola and Influenza (reviewed in [1]). In preventive HIV-1 vaccine development, three phase 2b, double-blind, placebo-controlled, randomized efficacy trials have been conducted to evaluate regimens containing recombinant Ad serotype 5 (rAd5) vector vaccines expressing HIV-1 antigens. The first two studies, Step [2] and Phambili [3], tested the MRKAd5 HIV-1 *gag/pol/nef* vaccine. Vaccinations in Step were stopped early due to efficacy futility observed at the first interim analysis, which caused the discontinuation of enrollment and vaccinations in Phambili. The third study, HVTN 505 (HVTN505) [4], tested a DNA prime- rAd5 HIV-1 *env/gag/pol* boost vaccine. Vaccinations in HVTN505 were also stopped early due to efficacy futility observed at the first interim analysis. Shortly after stopping of the vaccinations, participants of these studies were unblinded to their vaccination assignments but continued follow–up (Fig 1). Before the efficacy trials, three Phase 1–2 Triad studies, IAVI V001 (IAVI001) [5], RV 172 (RV172) [6] and HVTN 204 (HVTN204) [7], tested the same HVTN505 DNA/rAd5 vaccine regimen.

Post-hoc analysis of Step data revealed trends toward an increased risk of HIV-1 acquisition among vaccine recipients that waned over time, especially among males who had positive anti-Ad5 neutralizing antibodies (Ad5-positive) or were uncircumcised at baseline [2, 8]. Such a finding was not observed in the analyses of Phambili data shortly following study unblinding [3], nor of the long-term follow-up data [9].To clearly understand whether vaccine-associated increase of HIV-1 acquisition has occurred in one or more studies, we conducted a meta-analysis using up-to-date participant-level data from the efficacy trials and the three Triad studies. The objectives were to evaluate: 1) the effect of rAd5 vector vaccines on HIV-1 acquisition, and 2) variation in the effect across vaccine regimens, time since vaccination, and subgroups.

By combining information from multiple studies of similar interventions, a meta-analysis can provide clearer signals overall and in subgroups. However, this strength is predicated on the assumption that it is meaningful to combine data from multiple studies conducted in different populations with different Ad5 vector characteristics, expressing different HIV-1 antigens, and employing different vaccine regimens. In this individual participant data (IPD) meta-analysis, we obtained the raw individual level data from each study before they were combined for analyses, which is commonly referred to as individual patient data (IPD) meta-analysis when diseased study populations are involved. Compared to meta-analysis based on aggregated study-level summary data, IPD-based meta-analyses is usually more time-consuming but possesses many advantages, including but not limited to: 1) the statistical analysis can be standardized across studies; 2) complex relationships such as time-dependent effects can be modeled; 3) prognostic models (e.g., risk scores) can be generated and validated, and multiple individual level factors can be examined in combination; 4) estimates adjusted for baseline (prognostic) factors can be produced, which may increase statistical power and allow adjustment for potential confounding factors; and 5) meta-analysis results for specific subgroups of participants can be obtained across studies, and differential (treatment) effects can be assessed across individuals, which can help reduce between-study heterogeneity (e.g., [10, 11]).

## Methods

### Study selection and data extraction

Fred Hutchinson Cancer Research Center Institutional Review Board approved the described study. Consultation with HIV-1 vaccine developers and vaccine research experts, as well as a systematic literature search through February 2015 of the US National Library of Medicine (PubMed.gov), Ovid Medline (R) (1946-present) and clinicaltrials.gov was conducted to identify all randomized trials of Ad5-vectored preventive HIV-1 vaccines (Methods in S1 Supporting Information). Participant-level data were directly provided by the coordinating centers of the selected studies. A completed PRISMA (Preferred Reporting Items for Systematic Reviews and Meta-Analyses) checklist is provided in S2 Supporting Information.

### Individual-study features

The three Phase 2b trials enrolled participants at increased risk for HIV-1 infection in the Americas and Australia (Step), South Africa (Phambili) and the US (HVTN505); the three Phase 1–2 trials enrolled participants at low to intermediate risk for HIV-1 infection in East Africa (IAVI001 and RV172), and in the Americas and South Africa (HVTN204). With the exception of HVTN505, which enrolled circumcised men who have sex with men (MSM) and transgender women with no pre-existing anti-Ad5 antibodies (Ad5-negative), the studies enrolled men and non-pregnant women with no specific eligibility criteria based on baseline Ad5 serostatus or circumcision status (Table 1). Step and Phambili evaluated the MRKAd5 HIV-1 vaccine administered at weeks 0, 4 and 26 [2, 3]. HVTN505 and the Triad studies evaluated the 6-plasmid VRC-DNA vaccine administered at weeks 0, 4, and 8, followed by the VRC-rAd5 boost administered at week 24 [4]. The DNA placebo was phosphate-buffered saline and the rAd5-HIV placebo was vaccine diluent only [2–4]. The Phase 1–2 trials evaluated the same vaccines as HVTN505. Each individual protocol was approved by institutional review boards at all participating sites. All study participants provided written informed consent.

**Table 1:** Key Features of Individual Studies Included in the Meta-analysis.

	Step [2]	Phambili [3, 9]	HVTN505 [4]	IAVI001 [5]	RV172 [6]	HVTN204 [7]
**Vaccine regimen**	rAd5 (week 0), rAd5 (Week 4), rAd5 (week 26)		DNA (Week 0), DNA (Week 4), DNA (Week 8), rAd5 (Week 24)			
**Vaccine products**	The MRKAd5 HIV-1 gag/pol/nef vaccine consists of a 1:1:1 mixture of three separate replication-defective Ad5 vectors, one each expressing the gag gene from the HIV-1 strain CAM-1, the pol gene from HIV-1 strain IIIB, and the nef gene from HIV-1 strain JR-FL.		The DNA-HIV vaccine (VRC-HIVDNA-016-00-VP) consists of six DNA plasmid in equal concentrations that encode Gag from the clade B HIV-1 strain HXB2, Pol from the clade B HIV-1 strain NL4-3, and Nef from the clade B strain NY5/BRU, and HIV-1 Env glycoproteins from clade A (strain 92rw020), clade B (strain HXB2/BaL), and clade C (strain 97ZA012). The rAd5-HIV vaccine (VRC-HIVADV014-00-VP) consists of four rAd5 vectors in 3:1:1 ratio that encode the HIV-1 Gag-Pol polyprotein from clade B (strains HXB2-NL4-3) and HIV-1 Env glycoproteins from clades A, B and C matching the DNA Env components.			
**Vaccine dose**	1.5x10^10 viral genomes of MRKAd5		Four mg of VRC-DNA; 10^10 particle unit of VRC-Ad5	Four mg of VRC-DNA, 10^10 or 10^11 particle unit of VRC-Ad5		Same as HVTN505
**Placebo product**	Vaccine diluent with no Ad5 vector		Sterile phosphate buffered saline as DNA placebo; vaccine diluent with no Ad5 vector as Ad5 placebo			
**Delivery method**	Intramuscular injection of Ad5/placebo using a needle and syringe		Intramuscular injection of DNA/placebo using Bioinjector 2000 Needle-Free Injection System; intramuscular injection of Ad5/placebo using a needle and syringe			
**Study population**	High-risk, HIV-1 uninfected men who have sex with men and heterosexual men and non-pregnant women aged 18–50 years	High-risk predominantly heterosexual men and non-pregnant women aged 18–35 years	High-risk circumcised and Ad5-negative men or transgender women who have sex with men aged 18–50 years	Low risk men and non-pregnant women aged 18–50 years	Low or intermediate risk men and up-pregnant women aged 18–50 years	Low or intermediate risk men and non-pregnant women aged 18–50 years
**Study regions**	North America, the Caribbean, South America, and Australia	South Africa	United States	East Africa (Kenya, Rwanda)	East Africa (Uganda, Kenya, Tanzania)	The Americas (United States, Haiti, Jamaica and Brazil) and South Africa
**Randomization scheme**	1:1 (vaccine: placebo) randomization ratio pre-stratified by study site, sex, and baseline Ad5 antibody titer	1:1 (vaccine: placebo) randomization ratio pre-stratified by study site and sex	1:1 (vaccine: placebo) randomization ratio pre-stratified by study site	3:1 (vaccine: placebo) randomization ratio	1:1 (vaccine: placebo) randomization ratio for 180 participants; 2:1 ratio for the rest.	1:1 (vaccine: placebo) randomization ratio pre-stratified by geographical region
**Study enrollment period**	2004–2007	2007	2009–2013	2005–2006	2006	2006–2007
**Number of total randomized subjects**	3000	801	2530	79	281	466
Vaccine	1494	400	1264	58	158	233
Placebo	1506	401	1266	21	123	233
**Number of MITT subjects**	2970	800	2496	79	281	466
Vaccine	1478	400	1251	58	158	233
Placebo	1492	400	1245	21	123	233

### Meta-analysis endpoint and cohorts

The study endpoint was HIV-1 infection diagnosed during study follow-up. We predefined two co-primary analysis cohorts for assessing the vaccine effect on HIV-1 acquisition. The modified-intention-to-treat (MITT) cohort included all randomly assigned participants HIV-1 uninfected at study entry, who received at least the first vaccine/placebo, and the Ad5 cohort included MITT participants who received at least one dose of rAd5-HIV vaccine or rAd5-placebo. Post-randomization selection bias could possibly exist in the Ad5 cohort but not in the MITT cohort. The study included follow-up data through July 9, 2014.

### Statistical analysis

All reported meta-analyses were adjusted for baseline factors that could potentially confound the vaccine effect on HIV-1 infection, especially during the post-unblinding period. The following baseline factors were considered: region (North America + Australia vs. Other), age, sex, HSV-2 serostatus, race (White vs. Other), baseline Ad5 sero-positivity (Ad5-positive vs. Ad5-negative), self-reported circumcision status (circumcised vs. uncircumcised), and behavioral risk score. The behavioral risk score was developed to summarize the level of risk behaviors for men and women. It takes a value from 0 to 7 for men and from 0 to 6 for women. The risk score is defined as the number of high risk behaviors a subject self-reported during the 3–6 months prior to enrollment: for men 1) number of male partners >2, 2) use of any recreational or injected drug, 3) unprotected insertive anal sex, 4) unprotected receptive anal sex, 5) unprotected vaginal sex, 6) exchange sex for money, goods or services, 7) other sexually transmitted infections (STIs); and for women: 1) number of male partners >2, 2) use of any recreational or injected drug, 3) unprotected anal sex, 4) unprotected vaginal sex, 5) exchange sex, and 6) other STIs.

We measured the vaccine effect as one minus the hazard ratio (HR) of infection (vaccine: placebo) using multivariable Cox regression models stratified by study. To reduce bias due to inclusion of post-unblinding data, the Cox models adjusted for the aforementioned potential baseline confounding variables that best predicted infection based on all-subsets model selection and the exact Akaike’s information criterion (AIC). Grambsch and Therneau’s test [12] was applied to assess whether the adjusted HR was constant over time. For the co-primary analyses, the time-to-event variable was defined as the time from enrollment/initial vaccination (MITT cohort) or the first rAd5-HIV vaccination (Ad5 cohort) to the estimated time of HIV-1 infection. The estimated infection time, for the Phase 2b studies, was the midpoint between the last HIV-1 seronegative visit date and the first evidence of HIV-1 infection date based on both HIV-1 antibody and nucleic acid blood testing, and for the Phase 1–2 studies, was the date of HIV-1 diagnosis confirmed by ELISA, Western blot and RT-PCR. Participants who never showed any evidence of HIV-1 infection were right censored on the date of last HIV-1 test. For secondary analyses that restricted to data before unblinding or in the first 18 months following vaccination, we censored the follow-up time at the date of study unblinding or 18 months. We generated group-specific Kaplan-Meier (KM) curves, group difference of the cumulative probability of HIV-1 infection, and estimated non-parametric instantaneous HRs over time with 95% simultaneous confidence intervals [13].

We used Wald interaction tests based on Cox models to assess whether the vaccine effect differed by study, vaccine regimen, baseline covariates (sex, Ad5 serostatus, circumcision status), and time-varying covariates: time-period (follow-up time (FU) before or after 18 months since enrollment, i.e., FU≤18 months or FU>18 months) and number of rAd5-HIV vaccinations received (1, 2 or 3). Before looking into differences within a specific subset of studies or examining the effects of a specific vaccine regimen, we first conducted overall interaction tests to assess whether the vaccine effect differed across all six studies or all three efficacy trials, followed by interaction tests to assess whether the vaccine effect differed between the two vaccine regimens. Holm-Bonferroni family-wise-error-rate (FWER)-adjusted p-values and false-discovery-rate (FDR)-adjusted q-values were calculated to reflect the potential effect of multiple testing on type I errors. All reported p values were two-sided.

To investigate the power of this study in examining whether the two vaccine regimens were similar in terms of their effect on HIV-1 infection as compared to placebo recipient, we calculated the power for comparing different true HRs between Step+Phambili and HVTN505. The purpose of such power calculations was not to justify statistically non-significant results in a single study, but rather to provide an understanding of the capability of the meta-analysis in assessing whether several experiments (i.e., Step+Phambili vs. HVTN505) were similar [14]. The power calculations were based on the MITT cohort. Briefly, we fixed the total sample size, proportion of subjects randomly allocated to vaccine and placebo, and the number of HIV-1 infections in the placebo groups of HVTN505 and Step+Phambili. Specifically, for HVTN505, we fixed the sample size of 2496, with 1251 and 1245 MITT participants in the vaccine and placebo groups, respectively; we also fixed the number of HIV-1 infections in the placebo group to be 61. For Step+Phambili, we fixed the overall sample size of3770, with 1878 and 1892 MITT participants in the vaccine and placebo groups, respectively; we also fixed the number of HIV-1 infections in the placebo group to be 118. We studied a range of true HRs based on the 95% CI of the estimated HRs from Step+Phambili and HVTN505. Specifically, we chose the true HR of Step+Phambili to vary between 1.11 and 1.78, and the true HR for HVTN505 to vary between 0.47 and 1.26. For each pair of assumed true HRs, we computed the power for testing the equality in HRs between Step+Phambi and HVTN505 based on a Wald test statistic, assuming asymptotic normality of the difference in estimated log(HR) between the two groups; variance of the estimated log(HR) was computed using formula (10) in [15]. A 0.05-alpha level was used in the power calculations.

Because potential post-unblinding bias in the analysis could occur due to differential dropout, HIV-1 risk behavior or ascertainment of HIV-1 infection between vaccine and placebo recipients, pre-specified simulation-based sensitivity analyses were used to assess the impact of early unblinding in Step and Phambili. Simulations were also conducted to assess the impact of early stopping in HVTN505 on the reported HR estimates (Methods in S1 Supporting Information). Statistical analyses were performed with R version 2.15.1 [16]. An R function for generating the forest plots presented in this paper is included in the Supplement (Methods in S1 Supporting Information).

## Results

**Fig 2:**
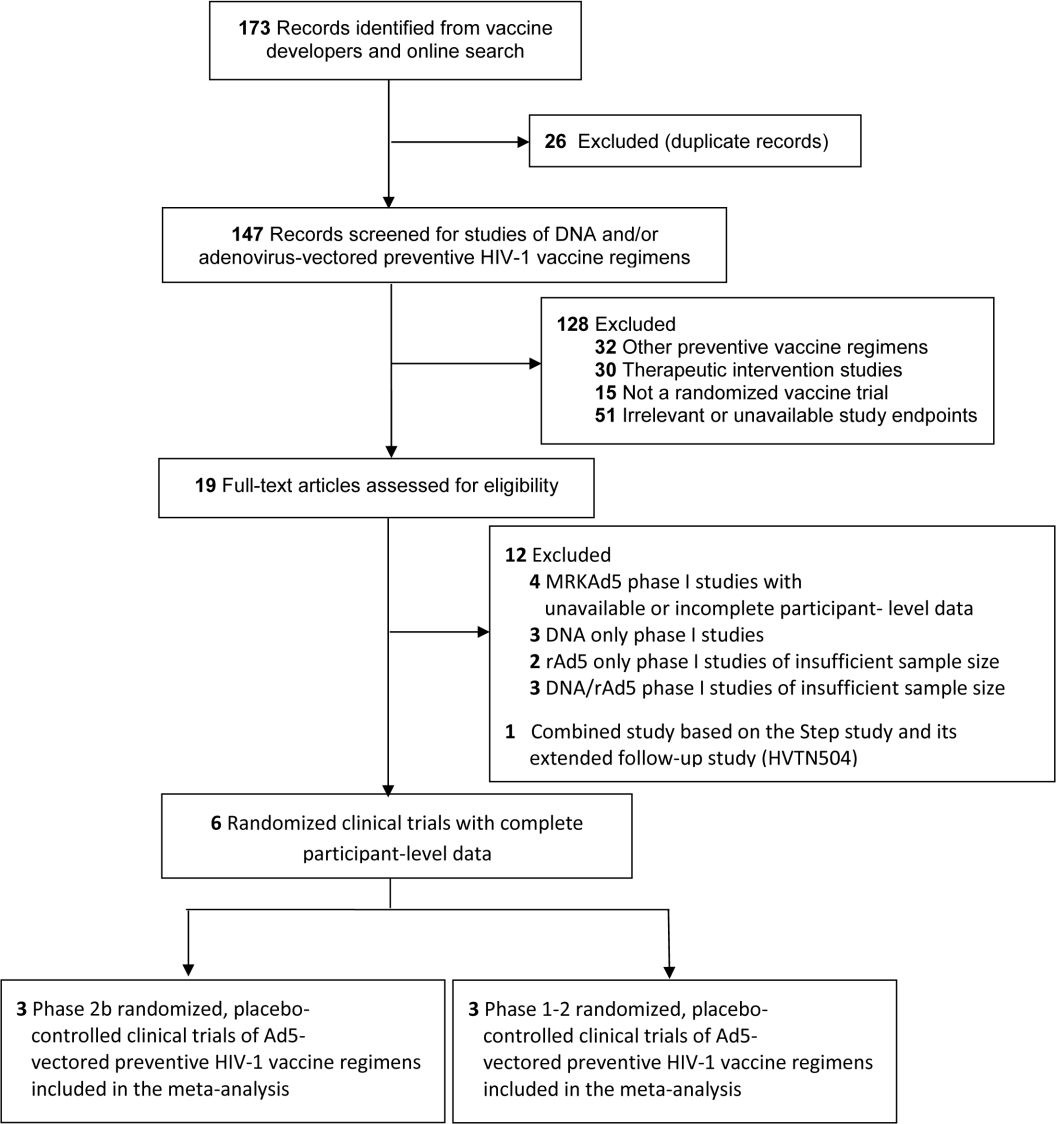
Study Flow Diagram.

We screened 173 articles for eligibility and identified 19 potentially relevant candidate HIV-1 vaccine studies for further review. Among the 19 studies, five evaluated DNA only [17–19] or rAd5 only [20, 21] vaccine regimens that are different from the ones evaluated in the efficacy trials; seven [22–28] evaluated the same vaccine regimens as the efficacy trials, but there were resource constraints in extracting the individual-level data from those trials. Given only a marginal number of HIV infections would be added to the total HIV infections beyond the three efficacy trials (< 6%), those 12 studies were excluded from the IPD meta-analysis. After also excluding one duplicate study [2], up-to-date participant-level data, some of which have not been previously published, from a total of six randomized, placebo-controlled trials of Ad5-vectored HIV-1 vaccines were included for the final meta-analysis (Fig 2). Table 2 provides key data summary of the six studies. All MITT participants in Step (n = 2970) and Phambili (n = 800) received at least one dose of rAd5-HIV vaccine or rAd5-placebo, whereas 82.8% of MITT participants in HVTN505 (n = 2496), 92.4% in IAVI001 (n = 79), 92.2% in RV172 (n = 281) and 89.1% in HVTN204 (n = 466) received a dose of rAd5-HIV vaccine or rAd5-placebo. The annual loss to follow-up incidence pooled over the vaccine and placebo groups was 10.2% in Step, 8.2% in Phambili and 10.8% in HVTN505. Table A in S1 Supporting Information shows the included information in the meta-analysis as compared to prior publications of the individual trials. Figs A and B in S1 Supporting Information show the covariate-unadjusted cumulative incidences of HIV-1 infection by treatment arm for the individual Phase 2b studies and Triad studies.

**Table 2:** Key Data Summary of Individual Studies Included in the Meta-analysis.

	Step[2]	Phambili [3, 9]	HVTN505 [4]	IAVI001 [5]	RV172 [6]	HVTN204 [7]
**Number of MITT subjects**	2970	800	2496	79	281	466
Vaccine	1478	400	1251	58	158	233
Placebo	1492	400	1245	21	123	233
**Percent of subjects received**						
0 Ad5/Placebo vaccinations	0%	0%	17.2%	8%	7%	10%
1 Ad5/Placebo vaccinations	3.0%	27.0%	82.8%	92.4%	92.2%	89.1%
2 Ad5/Placebo vaccinations	6.8%	66.1%	Not applicable (NA)	NA	NA	NA
3 Ad5/Placebo vaccinations	90.1%	6.9%	NA	NA	NA	NA
**Follow-up time since enrollment (median with range in months)**	39.1 (0, 51.1)	42.4 (0, 43.4)	25 (0, 58.5)	67.9 (5.4, 72.3)	55.3 (1.2, 74.8)	33.9 (0.4, 37.3)
Vaccine	39.2 (0, 50.3)	42.3 (0, 43.4)	25.9 (0, 58.3)	68.0 (5.4, 72.3)	56.7 (2, 70.6)	34.3 (0.4, 37.3)
Placebo	39.0 (0, 51.1)	42.4 (0, 43.4)	24.6 (0, 58.5)	67.9 (48.8,71.8)	54.2 (1.2, 74.8)	33.6 (0.9, 37.2)
**Number of infections: total, FU≤18 months, FU>18 months**	187, 93, 94	100, 51, 49	116, 78, 38	0, 0, 0	14, 9, 5	12, 8, 4
Vaccine	106, 55, 51	63, 29, 34	55, 40, 15	0, 0, 0	8, 4, 4	7, 5, 2
Placebo	81, 38, 43	37, 22, 15	61, 38, 23	0, 0, 0	6, 5, 1	5, 3, 2
**Number of infections by number of Ad5/placebo vaccinations: total, 0-Ad5/placebo, 1-Ad5/placebo, 2- Ad5/placebo, 3-Ad5/placebo**	187, 0, 1, 23, 163	100, 0, 25, 69, 6	116, 19, 97, 0, 0	0, 0, 0, 0, 0	14, 1, 13, 0, 0	12, 7, 5, 0, 0
Vaccine: total, 0-Ad5, 1-Ad5, 2 Ad5, 3-Ad5	106, 0, 0, 10, 96	63, 0, 18, 42, 3	55, 10, 45, 0, 0	0, 0, 0, 0, 0	8, 1, 7, 0, 0	7, 4, 3, 0, 0
Placebo: total, 0-placebo, 1-placebo, 2-placebo, 3-placebo	81, 0, 1, 13, 67	37, 0, 7, 27, 3	61, 9, 52, 0, 0	0, 0, 0, 0, 0	6, 0, 6, 0, 0	5, 3, 2, 0, 0
**Annual HIV-1 incidence (%): total, FU≤18 months, FU>18 months**	2.2, 2.3, 2.1	4.4, 4.8, 4.1	2.1, 2.4, 1.8	0, 0, 0	1.3, 2.4, 0.7	1.2, 1.3, 0.9
Vaccine	2.5, 2.8, 2.2	5.6, 5.5, 5.7	2, 2.4, 1.3	0, 0, 0	1.3, 1.8, 0.9	1.3, 1.7, 0.9
Placebo	1.9, 1.9, 1.9	3.2, 4.1, 2.4	2.3, 2.3, 2.2	0, 0, 0	1.3, 3.1, 0.3	1.0, 1.0, 1.0
**Annual loss to follow-up incidence (%), total, FU≤18 months, FU>18 months**	10.2, 7.9, 12.2	8.2, 8.4, 8.1	10.8, 10, 12.2			
Vaccine	10.0, 7.9, 11.8	7.8, 8.1, 7.6	9.8, 9.2, 10.6			
Placebo	10.4, 7.8, 12.7	8.6, 8.6, 8.6	11.9, 10.7, 13.9			
**Early unblinding date**	10/17/2007	10/23/2007	4/22/2013			
**Percent follow-up time at un-blinding**	47.3%	10.9%	67.30%			
Vaccine	47.0%	10.9%	66.80%			
Placebo	47.6%	10.9%	67.90%			
**Number of infections: total, pre-unblinding, post-unblinding**	187, 100, 87	100, 14, 86	116, 88, 28			
Vaccine	106, 57, 49	63, 8, 55	55, 46, 9			
Placebo	81, 43, 38	37, 6, 31	61, 42, 19			
**Annual HIV-1 incidence (%): total, pre-unblinding, post-unblinding**	2.2, 2.5, 1.9	4.4, 5.7, 4.3	2.1, 2.4, 1.6			
Vaccine	2.5, 2.9, 2.2	5.6, 6.5, 5.5	2.0, 2.5, 1.0			
Placebo	1.9, 2.1, 1.7	3.2, 4.8, 3.0	2.3, 2.3, 2.2			
**Annual loss to follow-up incidence (%): total, pre-unblinding, post-unblinding**	10.2, 8.0, 12.2	8.2, 8.1, 8.3	10.8, 11.4, 9.7			
Vaccine	10.0, 7.8, 11.9	7.8, 8.1, 7.8	9.8, 10.5, 8.4			
Placebo	10.4, 8.2, 12.5	8.6, 8.0, 8.7	11.9, 12.3, 11.1			

The pooled MITT cohort included 6266 participants (n = 3129 vaccine; n = 3137 placebo) from the three Phase 2b studies and 826 participants (n = 449 vaccine; n = 377 placebo) from the three Phase 1–2 studies. In the MITT cohort, 76.2% and 54.7% of the Phase 2b and Phase 1–2 study participants were men, respectively. Within each sex, baseline characteristics were balanced between the vaccine and placebo arms (Table B in S1 Supporting Information).

The pooled Ad5 cohort included 5824 participants (n = 2900 vaccine; n = 2924 placebo) from the three Phase 2b studies and 747 participants (n = 402 vaccine; n = 345 placebo) from the three Phase 1–2 studies, with balanced baseline characteristic between the vaccine and placebo arms. For simplicity, we report the remaining meta-analysis results excluding the Phase 1–2 studies, since over 91% of the HIV-1 infections occurred in the Phase 2b studies and both the overall and subgroup HR estimates were very similar when the Phase 1–2 studies were included (Fig C in S1 Supporting Information).

### Overall vaccine effect in Step, Phambili, and HVTN505

**Fig 3:**
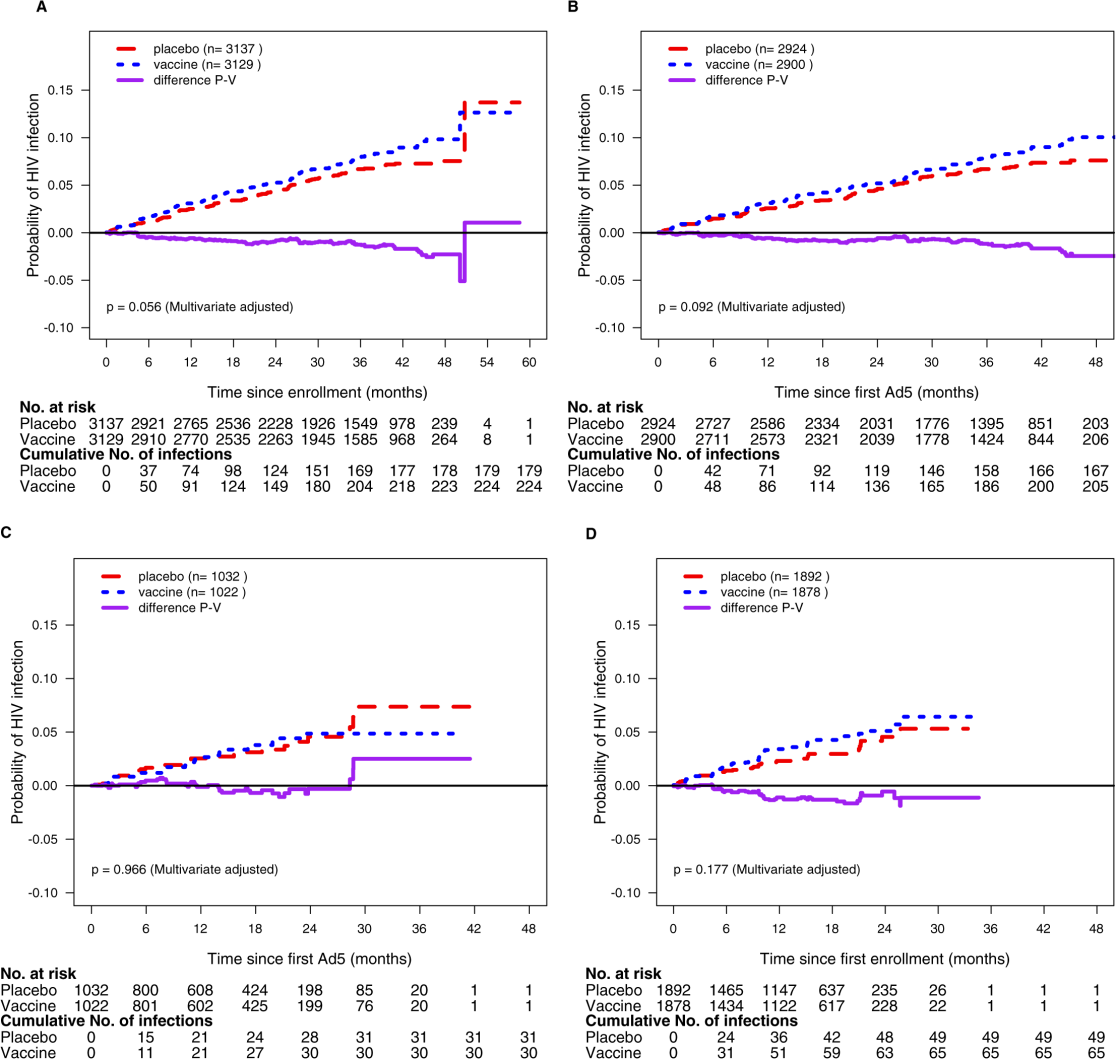
Kaplan-Meier Curves and Their Difference (Vaccine vs Placebo) of Cumulative Incidence of HIV-1 Infection. **These incidence estimates were not adjusted for covariates.** Panel A is for the MITT cohort and Panel B is for the Ad5 cohort based on Step, Phambili and HVTN505 combined using all follow-up time; Panel C is for the Ad5 Cohort based on HVTN505 alone using follow-up time before unblinding, and Panel D is based on Step and Phambili combined using follow-up time before unblinding. The MITT cohort included all randomly assigned participants HIV-1 uninfected at study entry, who received at least one dose of vaccine or placebo; the Ad5 cohort included MITT participants who received at least one dose of rAd5-HIV vaccine or rAd5-placebo.

**Fig 4:**
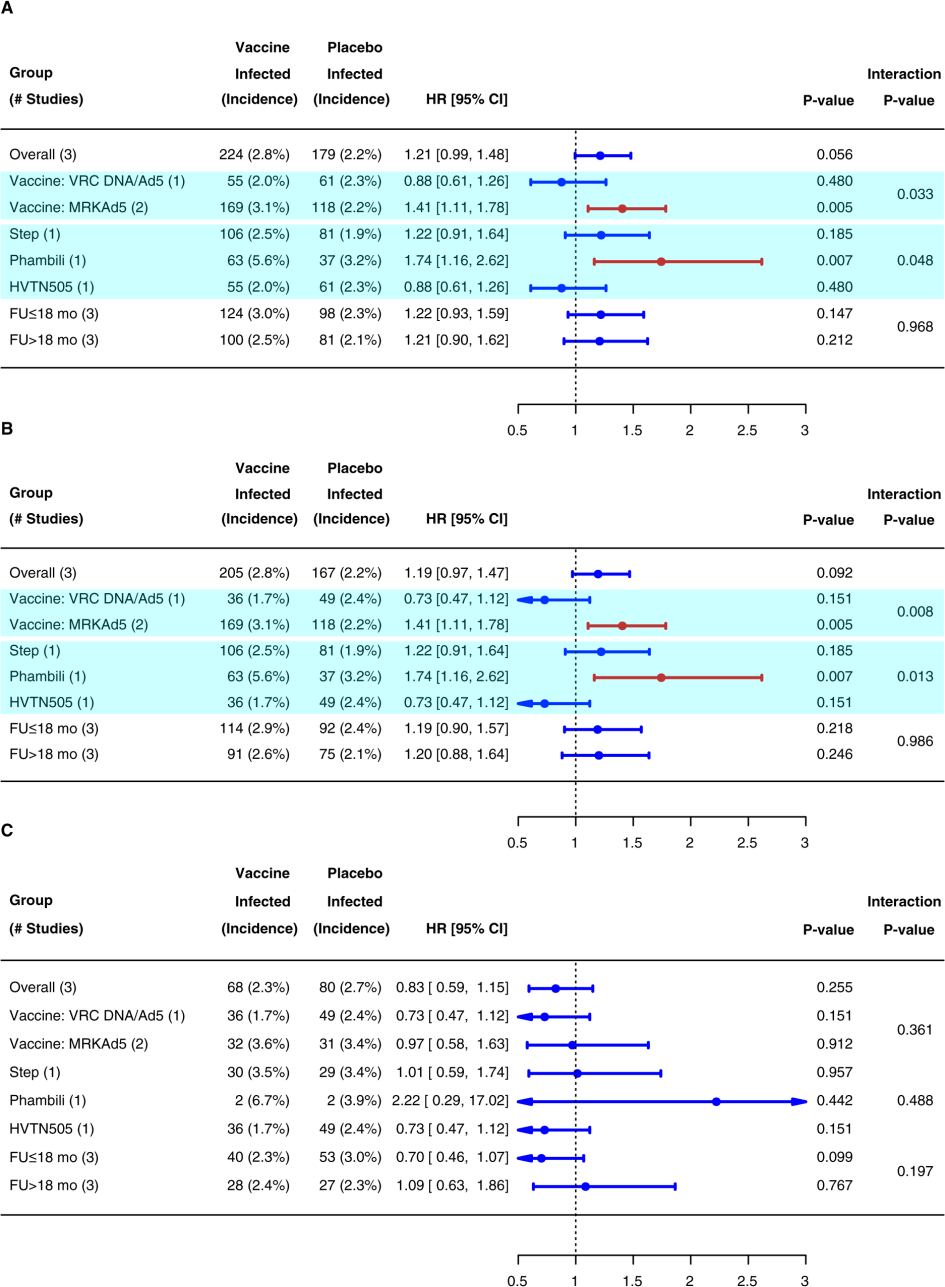
Estimated Covariate-Adjusted HRs of HIV-1 Infection (Vaccine vs Placebo) Based on Step, Phambili and HVTN505 Combined. Panel A is for the MITT Cohort, Panel B is for the Ad5 cohort, and Panel C is for circumcised Ad5-negative men in the Ad5 Cohort. The MITT cohort included all randomly assigned participants HIV-1 uninfected at study entry, who received at least one dose of vaccine or placebo; the Ad5 cohort included MITT participants who received at least one dose of rAd5-HIV vaccine or rAd5-placebo. Red lines are 95% confidence intervals indicating HRs significantly different from 1.0; blue lines are 95% confidence intervals indicating HRs not significantly different from 1.0. Shaded rows indicate significantly different HRs within subgroups.

The covariate-unadjusted cumulative incidences of HIV-1 infection in the MITT and Ad5 cohorts based on the phase 2b studies are plotted in Fig 3A and 3B. In the MITT cohort, 403 (n = 224 vaccine; n = 179 placebo) of 6266 participants acquired HIV-1, with a non-significantly higher incidence in vaccine recipients (HR 1.21, 95% CI 0.99–1.48, *P* = 0.06). In the Ad5 cohort, 372 (n = 205 vaccine; n = 167 placebo) of 5824 participants acquired HIV-1 (HR 1.19, 95% CI 0.97–1.47, *P* = 0.09). The overall interaction tests indicated that the HRs significantly differed between the three phase 2b studies in the MITT and Ad5 cohorts (*P* = 0.05 and 0.01, respectively), possibly due to the difference between the two vaccine regimens. Despite the limited power to detect HR differences (Fig D in S1 Supporting Information), interaction tests indicated that the HRs significantly differed by vaccine regimen in each of the MITT and Ad5 cohorts (*P* = 0.03 and 0.008, respectively (Fig 4A and 4B). Specifically, when we analyzed each vaccine regimen, the estimated HR for DNA/rAd5 in HVTN505 was 0.88 (95% CI 0.61–1.26, *P* = 0.48) in the MITT cohort and 0.73 (95% CI 0.47–1.12, *P* = 0.15) in the Ad5 cohort compared to 1.41 (95% CI 1.11–1.78, *P* = 0.005) in both cohorts for MRKAd5 in Step and Phambili combined, suggesting differences in the effect of the two vaccine regimens or in the study populations.

In studying the overall HR in different time periods pooling over the three efficacy trials, the overall HRs were almost identical by period of follow up (FU≤18 months vs. FU> 18 months) (interaction *P* > 0.90) (Fig 4), and the estimated HR over time in daily increments was fairly constant throughout follow-up (Fig E in S1 Supporting Information). An additional analysis showed similar HRs across subgroups who received one, two, or three rAd5-HIV vaccinations (Table C in S1 Supporting Information).

### Step and Phambili pooled

Additional analyses concerning the issue of unblinding and subgroups were then conducted in Step and Phambili combined. HVTN505 was excluded based on several considerations. First, Step and Phambili tested the same MRKAd5 vaccine whereas HVTN505 tested a different vaccine. Second, the HRs differed significantly between the two vaccine regimens as shown above. Third, Step and Phambili comprised about 70% of the total HIV-1 infections and about 90% of the infections diagnosed after 18 months of follow-up. Lastly, Step and Phambili included Ad5-positive participants, uncircumcised men, and women, groups that were ineligible for HVTN505.

**Fig 5:**
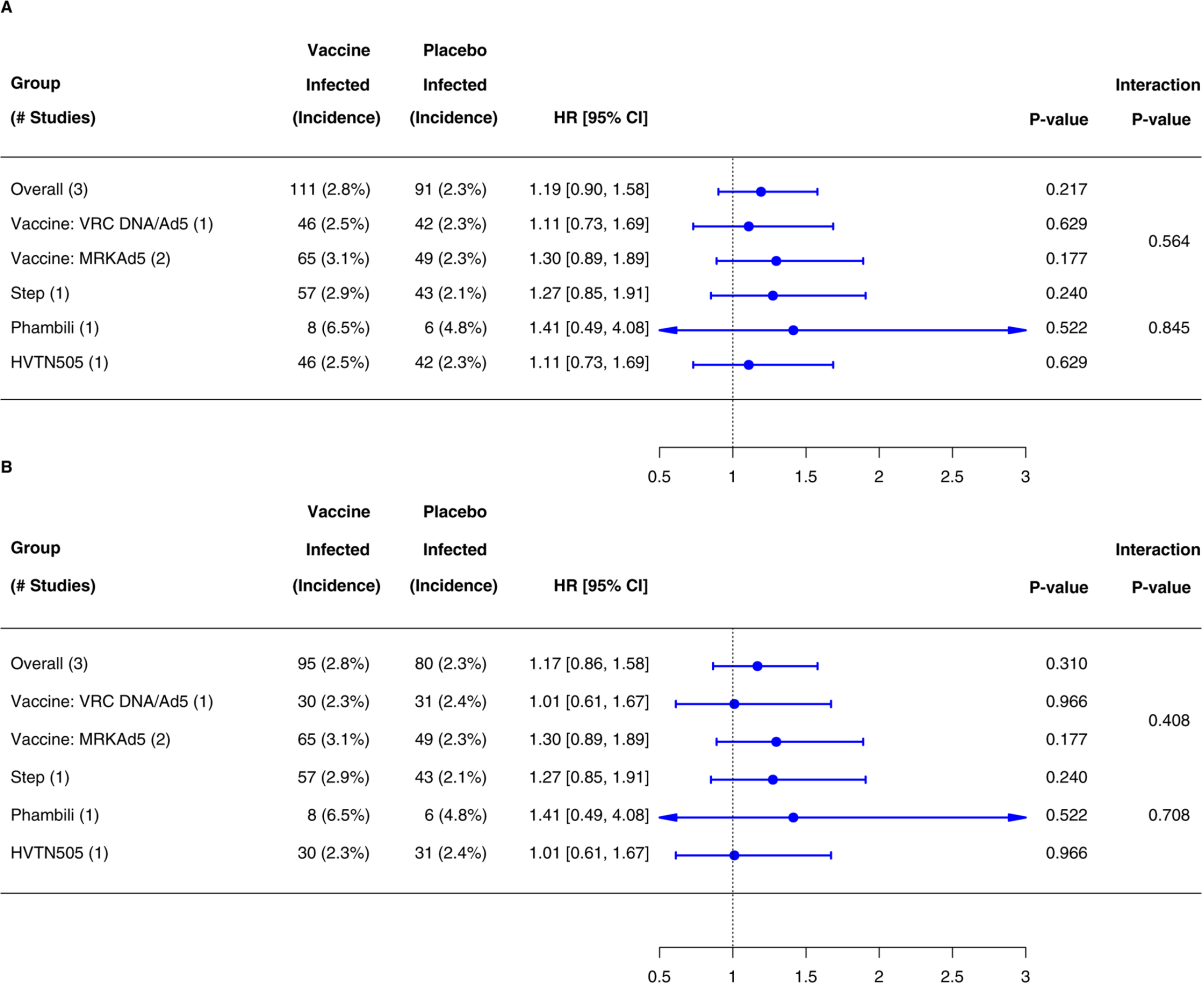
Estimated HRs in the MITT Cohort (Panel A) and Ad5 Cohort (Panel B) Using Follow-up Time before Unblinding Based on Step, Phambili and HVTN505. The MITT cohort included all randomly assigned participants HIV uninfected at study entry, who received at least one dose of vaccine or placebo; the Ad5 cohort included MITT participants who received at least one dose of rAd5-HIV vaccine or rAd5-placebo. Blue lines are 95% confidence intervals indicating HRs not significantly different from 1.0.

When restricting the analysis of Step and Phambili to follow-up time before unblinding where balance in HIV-1 risk factors between the vaccine and placebo groups is most assured, 114 (n = 65 vaccine; n = 49 placebo) of 3770 MITT participants acquired HIV-1, with a non-significantly higher incidence in MRKAd5 vaccine recipients (HR 1.30, 95% CI 0.89–1.14, P = 0.18) (Figs 3D and 5). Additional sensitivity analyses explored the impact of potential bias in the post-unblinding period (Methods, Table D and Fig F in S1 Supporting Information).

Comparing Step and Phambili, there was no strong evidence of a differential vaccine effect when including all follow-up, restricting to FU≤18 months, or restricting to FU> 18 months (Fig G in S1 Supporting Information). Similar HRs were also observed among subgroups defined by sex, baseline Ad5 serostatus or baseline circumcision status when evaluated over the entire duration of follow-up (all interaction *P*>0.30) (Fig H(A) in S1 Supporting Information). Of note, when restricting the analysis to circumcised and Ad5-seronegative men, the study population participating in HVTN505, the estimated HR was 0.97 (95% CI 0.58–1.63, *P* = 0.91) in Step and Phambili combined (Fig C and Fig I in S1 Supporting Information), supporting that neither vaccine regimen increased HIV-1 risk in this subgroup.

We next analyzed the vaccine effect by follow-up time within each participant subgroup (Figs H, J, and K in S1 Supporting Information). Statistical tests of interaction suggested a differential vaccine effect by duration of follow-up and baseline circumcision status (3-way interaction *P* = 0.002). Uncircumcised men had a vaccine-increased risk of HIV-1 infection before 18 months of enrollment (HR 2.90, 95% CI 1.49–5.65, *P* = 0.002), whereas circumcised men had a vaccine-increased risk after 18 months (HR 2.08, 95% CI 1.17–3.68, *P* = 0.01). Similarly, the vaccine-increased risk varied by duration of follow-up and baseline Ad5 serostatus (3-way interaction *P* = 0.004). Ad5-negative participants had a significant vaccine-increased risk only after 18 months (HR 1.92, 95% CI 1.07–3.46, *P* = 0.03); the HRs for this group differed significantly before and after 18 months (interaction *P* = 0.01). Ad5-positive participants had an early increased risk (HR 1.92, 95% CI 1.25–2.93, *P* = 0.003); however, the HRs for these participants did not significantly differ by follow-up time period. Of the 24 interaction tests conducted, one had an FWER-adjusted p-value < 0.05 and 11 had FDR-adjusted q-values < 0.20, supporting that several of the differential vaccine effect results are likely to be real (Table 3).

**Table 3:** Unadjusted and Multiplicity-Adjusted P-values from Interaction Tests of Differential Vaccine Effect by Follow-up Time Period and Subgroup Based on Step and Phambili. Holm-Bonferroni family-wise-error-rate (FWER)-adjusted p-values and false-discovery-rate (FDR)-adjusted q-values are reported in the last two columns.

Follow-up Time Period	Factor (Subgroup)	Unadjusted Interaction P-values	FWER-adjusted P-values	FDR-adjusted Q-values
Overall	Study (Step vs Phambili)	0.21	1.0	0.37
	Gender (Male vs Female)	0.62	1.0	0.79
	Ad5 serostatus (positive vs negative)	0.24	1.0	0.39
	Ad5 serostatus in men (positive vs negative)	0.37	1.0	0.55
	Circumcision status (yes vs no)	0.54	1.0	0.77
FU ≤ 18 months	Study (Step vs Phambili)	0.92	1.0	0.96
	Gender (Male vs Female)	0.84	1.0	0.92
	Ad5 serostatus (positive vs negative)	0.004	0.09	0.03
	Ad5 serostatus in men (positive vs negative)	0.002	0.05	0.03
	Circumcision status (yes vs no)	0.01	0.21	0.05
FU > 18 months	Study (Step vs Phambili)	0.06	0.96	0.15
	Gender (Male vs Female)	0.61	1.0	0.79
	Ad5 serostatus (positive vs negative)	0.21	1.0	0.37
	Ad5 serostatus in men (positive vs negative)	0.05	0.83	0.13
	Circumcision status (yes vs no)	0.08	1.0	0.18
FU ≤ 18 months vs FU > 18 months	All participants	0.70	1.0	0.80
	Female	0.96	1.0	0.96
	Male	0.68	1.0	0.80
	Ad5-negative	0.01	0.21	0.05
	Ad5-positive	0.16	1.0	0.32
	Ad5-negative men	0.003	0.07	0.03
	Ad5-positive men	0.05	0.83	0.13
	Circumcised men	0.02	0.40	0.08
	Uncircumcised men	0.04	0.77	0.13

## Discussion

This meta-analysis of six randomized, double-blind, placebo-controlled clinical studies and their long-term follow-up in 7092 participants is the first of its kind to systematically evaluate the effect of rAd5-vectored HIV-1 vaccines on susceptibility to HIV-1 acquisition. Based on rigorous statistical analysis of pooled individual participant-level data, we did not observe statistically significant evidence of increased risk of HIV-1 infection among vaccine recipients when all study participants and follow-up time were considered. We observed that vaccine-associated risk of HIV-1 infection significantly differed by vaccine regimen and/or study population: there was evidence of increased risk for Step and Phambili that tested the MRKAd5 vaccine in both men and women, but no evidence of vaccine-increased risk when the VRC-DNA/rAd5 vaccine was analyzed by itself (using data from circumcised Ad5-negative men in HVTN505, and men and women in the Triad studies). When restricting to follow-up while the studies were blinded, the evidence for increased risk was not statistically significant for all studies combined or separately for either vaccine regimen. When subgroups were examined separately over early and late follow-up, nominally statistically significant evidence was observed for early increased risk among uncircumcised men, Ad5-positive participants and Ad5-positive men, and for late increased-risk among circumcised men, Ad5-negative participants and Ad5-negative men.

By aggregating data, meta-analysis provides statistical power to discover signals that individual studies were under-powered to assess. Our meta-analysis demonstrated a significantly increased risk of HIV-1 acquisition among MRKAd5 vaccine recipients who entered the trials with evidence of past Ad5 infection; this effect appeared not to mitigate after long term follow-up. In addition, among MRKAd5 vaccine recipients without evidence of past Ad5 infection or who were circumcised men, significantly higher risk was seen during long term follow-up. These observations were not previously reported in the individual studies. It is uncertain whether these observations were confounded by potential imbalances in HIV exposure between the vaccine and placebo groups in the late time period due to unblinding, although the likelihood of such confounding reversing the inference of increased risk appeared to be low based on our sensitivity analyses. Previous studies in Step participants showed that pre-existing Ad immunity could affect the magnitude and nature of vaccine-induced immune responses [29, 30]. However, whether Ad-specific or other vaccine-induced immune responses are related to differences in risk of HIV-1 acquisition after vaccination is unclear.

By aggregating data, meta-analysis also enables answering questions across study products and/or study populations that cannot be feasibly addressed with individual studies. Our meta-analysis showed that vaccine-associated risk of HIV-1 infection differed significantly between the MRKAd5 and DNA/rAd5 vaccine regimens when including all study participants and all follow-up time. When restricting to circumcised Ad5-negative men, the study population that provided the majority of infection data for DNA/rAd5, the vaccine effect on HIV-1 infection did not seem to differ between the two vaccine regimens, and there was no evidence of increased risk for either regimen. When restricting to follow-up while the studies were blinded, the vaccine effect did not seem to differ, either. Although the lack of evidence in both subgroups could be due to the limited statistical power to detect vaccine effect differences with a smaller number of infections, the latter analyses circumvented the issue of potential post-unblinding confounding by measured or unmeasured factors associated with HIV-1 infection risk. These findings suggest that differences in HIV antigen designs could have played a role in the different vaccine effects of MARKAd5 and DNA/rAd5; however, it is prudent to address such a hypothesis in the appropriate study populations and/or follow-up timeframe.

Because none of the trials used an empty rAd5 vector as a control arm, this meta-analysis cannot parse out if the vaccine-associated increased risk was due to the rAd5 vector or the HIV-1 vaccine inserts. A non-human primate challenge trial of a rAd5-vectored SIV vaccine mimicking the MRKAd5 vaccine addressed the vector versus insert question by using a control group of empty rAd5 vector immunized animals [31]. Although this trial recapitulated the result in Step of vaccine-increased risk, implicating the SIV vaccine inserts as the cause, it is unclear whether this result would apply to HIV-1 vaccines. We hypothesize that the HIV antigen design and the HIV-specific immune response patterns are more likely to influence susceptibility to HIV than the transient and non-specific immune stimulation of a vaccine vector.

This meta-analysis has several limitations. First, the selected trials tested two different rAd5-vectored vaccine regimens with different biological properties in different geographic regions and study populations. Therefore, the ability to interpret the results assumes that it is meaningful to combine data across the multiple interventions and settings that were included. Second, this study, like most meta-analyses, was not prospectively planned. Therefore, findings from this study should not be regarded as additional evidence from independent experiments, but rather integrated information across existing studies. Third, this study provided limited power for tests of different vaccine effects due to small numbers of infections in some subgroups, limited long-term follow-up prior to unblinding, and limited long-term follow-up in HVTN505. Fourth, unblinding of trial participants may have introduced bias in the analysis. Differential dropout, risk behavior and/or ascertainment of HIV-1 infection between treatment groups could have occurred after unblinding. To reduce such bias, all analyses controlled for baseline measured factors potentially prognostic for HIV-1 whose distributions could differ between treatment groups after unblinding. Future analysis may be conducted to provide causal vaccine effect estimates adjusting for prognostic factors (e.g. risk behavior) over time. The sensitivity analyses presented in the Supplement quantify the degree of such bias due to both measured and unmeasured confounding that could lead to reversing a conclusion of vaccine-increased risk. Lastly, early stopping of HVTN505 may cause the estimate of overall vaccine effect to be biased, although a bias-corrected analysis of HVTN505 showed only a small influence of early stopping (Methods and Fig L in S1 Supporting Information).

This study provides evidence for increased risk associated with the MRKAd5 vaccine overall and in subgroups except the circumcised and Ad5-negative men. While this meta-analysis does not provide a reliable basis for predicting whether rAd5-vectored vaccines for other pathogens or other rAd-vectored vaccines for HIV-1 would increase susceptibility to infection in HIV-1 at-risk populations, for large efficacy trials of such vaccines it provides a rationale for adding monitoring plans enabling detection of such increased susceptibility. Further research is needed to understand which attributes of the MRKAd5 vaccine regimen should be avoided in future HIV-1 vaccine design.

## Supporting Information

S1 Supporting Information Literature Search; R Function for Generating Forest Plots; Sensitivity Analysis of the Impact of Early Unblinding on Hazard Ratio (HR) Estimates; Analysis of Bias from Early Stopping in HVTN505 (Methods). Comparisons of Follow-up Time and Number of HIV-1 Infections between the Meta-analysis and Prior Publications of Individual Trials **(Table A).** Participant Baseline Characteristics (**Table B**). Estimates of Time-dependent Ad5-dose Response Based on Step and Phambili, with and without Including HVTN505 in the Ad5 Cohort (Table C). Sensitivity Analysis Example Scenario Based on Step and Phambili **(Table D).** Kaplan-Meier Curves and Their Difference of Cumulative Incidence of HIV-1 Infection for Individual Studies: Step, Phambili, HVTN505 and the Triad studies in the MITT Cohort **(Fig A).** Kaplan-Meier Curves and Their Difference of Cumulative Incidence of HIV-1 Infection for Individual Studies: Step, Phambili, HVTN505 and the Triad studies in the Ad5 Cohort **(Fig B).** Estimated HRs in the MITT Cohort (Panel A) and Ad5 Cohort (Panel B) Based on All Six Studies **(Fig C).** Power Calculations for Vaccine Effect Comparisons between HVTN505 vs. Step and Phambili Combined Based on Sample Sizes in the MITT Cohort (Panel A) and the Ad5 Cohort (Panel B) **(Fig D).** Nonparametric Instantaneous HR of Infection in the MITT Cohort (Panel A) and the Ad5 Cohort (Panel B) Based on Step, Phambili and HVTN505 **(Fig E).** Sensitivity Analysis to Assess the Impact of Post-unblinding Bias Based on Step and Phambili Combined **(Fig F).** Estimated HRs by Study and Follow-up Time Period Based on Step and Phambili **(Fig G).** Estimated HRs in Subgroups Using Follow-up Time Overall (Panel A), before 18 Months (Panel B) and after 18 Months (Panel C) Based on Step and Phambili **(Fig H).** Estimated HRs in the MITT Cohort (Panel A) and the Ad5 Cohort (Panel B) among Circumcised Ad5-negative Men Based on Step, Phambili and HVTN505 **(Fig I).** Estimated HRs by Follow-up Time Period in Subgroups Defined by Baseline Circumcision Status and Ad5 Serostatus Based on Step and Phambili **(Fig J)**. Summary of Estimated HRs and Interaction Test P-values by Follow-up Time Period and by Subgroups Defined by Baseline Circumcision Status and Ad5 Serostatus Based on Step and Phambili **(Fig K)**. Bias-corrected Relative Risk Accounting for Early Stopping in HVTN505 **(Fig L)**.
(DOCX)

S2 Supporting Information PRISMA Checklist. (DOCX)
