# Supplementary material for: Effect of rAd5-Vector HIV-1 Preventive Vaccines on HIV-1 Acquisition: A Participant-Level Meta-Analysis of Randomized Trials
Source: PLoS One. 2015 Sep 2;10(9):e0136626. doi: 10.1371/journal.pone.0136626 (PMC4558095; doi:10.1371/journal.pone.0136626)
Supplement: S1 Supporting Information — Comparisons of Follow-up Time and Number of HIV-1 Infections between the Meta-analysis and Prior Publications of Individual Trials (Table A). Participant Baseline Characteristics (Table B). Estimates of Time-dependent Ad5-dose Response Based on Step and Phambili, with and without Including HVTN505 in the Ad5 Cohort (Table C). Sensitivity Analysis Example Scenario Based on Step and Phambili (Table D). Kaplan-Meier Curves and Their Difference of Cumulative Incidence of HIV-1 Infection for Individual Studies: Step, Phambili, HVTN505 and the Triad studies in the MITT Cohort (Fig A). Kaplan-Meier Curves and Their Difference of Cumulative Incidence of HIV-1 Infection for Individual Studies: Step, Phambili, HVTN505 and the Triad studies in the Ad5 Cohort (Fig B). Estimated HRs in the MITT Cohort (Panel A) and Ad5 Cohort (Panel B) Based on All Six Studies (Fig C). Power Calculations for Vaccine Effect Comparisons between HVTN505 vs. Step and Phambili Combined Based on Sample Sizes in the MITT Cohort (Panel A) and the Ad5 Cohort (Panel B) (Fig D). Nonparametric Instantaneous HR of Infection in the MITT Cohort (Panel A) and the Ad5 Cohort (Panel B) Based on Step, Phambili and HVTN505 (Fig E). Sensitivity Analysis to Assess the Impact of Post-unblinding Bias Based on Step and Phambili Combined (Fig F). Estimated HRs by Study and Follow-up Time Period Based on Step and Phambili (Fig G). Estimated HRs in Subgroups Using Follow-up Time Overall (Panel A), before 18 Months (Panel B) and after 18 Months (Panel C) Based on Step and Phambili (Fig H). Estimated HRs in the MITT Cohort (Panel A) and the Ad5 Cohort (Panel B) among Circumcised Ad5-negative Men Based on Step, Phambili and HVTN505 (Fig I). Estimated HRs by Follow-up Time Period in Subgroups Defined by Baseline Circumcision Status and Ad5 Serostatus Based on Step and Phambili (Fig J). Summary of Estimated HRs and Interaction Test P-values by Follow-up Time Period and by Subgroups Defined by Baseline Circumcision Status and Ad [file pone.0136626.s001.docx]

**S1 Supporting Information**

Yunda Huang, Dean Follmann, Martha Nason, Lily Zhang, Ying Huang, Devan V. Mehrotra, Zoe Moodie, Barbara Metch, Holly Janes, Michael C. Keefer, Gavin Churchyard, Merlin L. Robb, Patricia E. Fast, Ann Duerr, M. Juliana McElrath, Lawrence Corey, John R. Mascola, Barney S. Graham, Magdalena E. Sobieszczyk, James G. Kublin, Michael Robertson, Scott M. Hammer, Glenda E. Gray, Susan P. Buchbinder, Peter B. Gilbert. *Effect of rAd5-vector HIV-1 Preventive Vaccines on HIV-1 Acquisition: a Participant-Level Meta-Analysis of Randomized Trials.*

**Methods: Literature Search**

**Methods: R Function for Generating Forest PlotseMethods: Sensitivity Analysis of the Impact of Early Unblinding on Hazard Ratio (HR) Estimates**

**Methods: Analysis of Bias from Early Stopping in HVTN505**

**Table Legends**

**Table A. Comparisons of Follow-up Time and Number of HIV-1 Infections between the Meta-analysis and Prior Publications of Individual Trials.**

**Table B. Participant Baseline Characteristics.**

**Table C. Estimates of Time-dependent Ad5-dose Response Based on Step and Phambili, with and without Including HVTN505 in the Ad5 Cohort.**

**Table D. Sensitivity Analysis Example Scenario Based on Step and Phambili.**

**Figure Legends**

**Figure A. Kaplan-Meier Curves and their Difference of Cumulative Incidence of HIV-1 Infection for Individual Studies: Step, Phambili, HVTN505 and the Triad studies in the MITT Cohort.**

**Figure B. Kaplan-Meier Curves and their Difference of Cumulative Incidence of HIV-1 Infection for Individual Studies: Step, Phambili, HVTN505 and the Triad studies in the Ad5 Cohort.**

**Figure C. Estimated HRs in the MITT Cohort (Panel A) and Ad5 Cohort (Panel B) Based on All Six Studies.**

**Figure D**. **Power Calculations for Vaccine Effect Comparisons between HVTN505 vs. Step and Phambili Combined Based on Sample Sizes in the MITT Cohort (Panel A) and the Ad5 Cohort (Panel B).**

**Figure E. Nonparametric Instantaneous HR of Infection in the MITT Cohort (Panel A) and the Ad5 Cohort (Panel B) Based on Step, Phambili and HVTN505.**

**Figure F. Sensitivity Analysis to Assess the Impact of Post-unblinding Bias in Step and Phambili Combined.**

**Figure G. Estimated HRs by Study and Follow-up Time Period Based on Step and Phambili.**

**Figure H. Estimated HRs in Subgroups Using Follow-up Time Overall (Panel A), before 18 Months (Panel B) and after 18 Months (Panel C) Based on Step and Phambili.**

**Figure I. Estimated HRs in the MITT Cohort (Panel A) and Ad5 Cohort (Panel B) among Circumcised Ad5-negative Men Based on Step, Phambili and HVTN505.**

**Figure J. Estimated HRs by Follow-up Time Period in Subgroups Defined by Baseline Circumcision Status and Ad5 Serostatus Based on Step and Phambili.**

**Figure K. Summary of Estimated HRs and Interaction Test P-values by Follow-up Time Period and by Subgroups Defined by Baseline Circumcision Status and Ad5 Serostatus Based on Step and Phambili.**

**Figure L. Bias-corrected Relative Risk Accounting for Early Stopping in HVTN505.**

**Methods: Literature Search**

**Database: clinicaltrials.gov**

Search criteria: adenovirus HIV vaccine | Interventional Studies | Phase 1, 2, 3, 4 | NIH, U.S. Fed, Industry, Other (47 records)

- [Acquired Immunodeficiency Syndrome](https://www.clinicaltrials.gov/ct2/results?term=adenovirus+HIV+vaccine&type=Intr&cond=%22Acquired+Immunodeficiency+Syndrome%22&phase=0123&fund=0123)   44 studies
- [Adenoviridae Infections](https://www.clinicaltrials.gov/ct2/results?term=adenovirus+HIV+vaccine&type=Intr&cond=%22Adenoviridae+Infections%22&phase=0123&fund=0123)   22 studies
- [Communicable Diseases](https://www.clinicaltrials.gov/ct2/results?term=adenovirus+HIV+vaccine&type=Intr&cond=%22Communicable+Diseases%22&phase=0123&fund=0123)   42 studies
- [DNA Virus Infections](https://www.clinicaltrials.gov/ct2/results?term=adenovirus+HIV+vaccine&type=Intr&cond=%22DNA+Virus+Infections%22&phase=0123&fund=0123)   1 study
- [HIV Infections](https://www.clinicaltrials.gov/ct2/results?term=adenovirus+HIV+vaccine&type=Intr&cond=%22HIV+Infections%22&phase=0123&fund=0123)   44 studies
- [HIV Seropositivity](https://www.clinicaltrials.gov/ct2/results?term=adenovirus+HIV+vaccine&type=Intr&cond=%22HIV+Seropositivity%22&phase=0123&fund=0123)   1 study
- [Immunologic Deficiency Syndromes](https://www.clinicaltrials.gov/ct2/results?term=adenovirus+HIV+vaccine&type=Intr&cond=%22Immunologic+Deficiency+Syndromes%22&phase=0123&fund=0123)   36 studies
- [Infection](https://www.clinicaltrials.gov/ct2/results?term=adenovirus+HIV+vaccine&type=Intr&cond=%22Infection%22&phase=0123&fund=0123)   42 studies
- [Influenza in Birds](https://www.clinicaltrials.gov/ct2/results?term=adenovirus+HIV+vaccine&type=Intr&cond=%22Influenza+in+Birds%22&phase=0123&fund=0123)   1 study
- [Influenza, Human](https://www.clinicaltrials.gov/ct2/results?term=adenovirus+HIV+vaccine&type=Intr&cond=%22Influenza%2C+Human%22&phase=0123&fund=0123)   1 study
- [Lentivirus Infections](https://www.clinicaltrials.gov/ct2/results?term=adenovirus+HIV+vaccine&type=Intr&cond=%22Lentivirus+Infections%22&phase=0123&fund=0123)   35 studies
- [Myelofibrosis](https://www.clinicaltrials.gov/ct2/results?term=adenovirus+HIV+vaccine&type=Intr&cond=%22Myelofibrosis%22&phase=0123&fund=0123)   1 study
- [Orthomyxoviridae Infections](https://www.clinicaltrials.gov/ct2/results?term=adenovirus+HIV+vaccine&type=Intr&cond=%22Orthomyxoviridae+Infections%22&phase=0123&fund=0123)   1 study
- [RNA Virus Infections](https://www.clinicaltrials.gov/ct2/results?term=adenovirus+HIV+vaccine&type=Intr&cond=%22RNA+Virus+Infections%22&phase=0123&fund=0123)   37 studies
- [Respiratory Tract Diseases](https://www.clinicaltrials.gov/ct2/results?term=adenovirus+HIV+vaccine&type=Intr&cond=%22Respiratory+Tract+Diseases%22&phase=0123&fund=0123)   1 study
- [Respiratory Tract Infections](https://www.clinicaltrials.gov/ct2/results?term=adenovirus+HIV+vaccine&type=Intr&cond=%22Respiratory+Tract+Infections%22&phase=0123&fund=0123)   1 study
- [Retroviridae Infections](https://www.clinicaltrials.gov/ct2/results?term=adenovirus+HIV+vaccine&type=Intr&cond=%22Retroviridae+Infections%22&phase=0123&fund=0123)   35 studies
- [Sexually Transmitted Diseases](https://www.clinicaltrials.gov/ct2/results?term=adenovirus+HIV+vaccine&type=Intr&cond=%22Sexually+Transmitted+Diseases%22&phase=0123&fund=0123)   35 studies
- [Sexually Transmitted Diseases, Viral](https://www.clinicaltrials.gov/ct2/results?term=adenovirus+HIV+vaccine&type=Intr&cond=%22Sexually+Transmitted+Diseases%2C+Viral%22&phase=0123&fund=0123)   35 studies
- [Syndrome](https://www.clinicaltrials.gov/ct2/results?term=adenovirus+HIV+vaccine&type=Intr&cond=%22Syndrome%22&phase=0123&fund=0123)   36 studies
- [Vaccinia](https://www.clinicaltrials.gov/ct2/results?term=adenovirus+HIV+vaccine&type=Intr&cond=%22Vaccinia%22&phase=0123&fund=0123)   2 studies
- [Virus Diseases](https://www.clinicaltrials.gov/ct2/results?term=adenovirus+HIV+vaccine&type=Intr&cond=%22Virus+Diseases%22&phase=0123&fund=0123)   39 studies

Database: Ovid MEDLINE(R) 1946 to February Week 1 2015

Search criteria: ((adenovirus or ad5 or DNA) and HIV and vaccine and human and clinical trial).af. (113 records)

Database: PubMed.gov

Search criteria: (((adenovirus[Title/Abstract]) OR ad5[Title/Abstract]) OR DNA[Title/Abstract]) AND HIV[Title/Abstract] AND Vaccine NOT ("animals"[MeSH Terms] NOT "humans"[MeSH Terms]): clinical trials (337 records)

**Methods: R Function for Generating Forest Plots**

The following R function outputs a forest plot as shown in the paper based on pre-calculated estimates of vaccine effect, corresponding standard errors and p-values. Extra columns can be included on the left of the barplots depicting descriptive statistics of each subgroup or row; extra columns can also be included on the right depicting the interaction p-values of treatment effect across multiple subgroups or rows. Note that texts following the # sign are comments.

# ##############################################################

# Author: Lily Zhang @ Fred Hutchinson Cancer Research Center

# Date: 9/9/2013

#################################################################

##############

# Input Parameters:

# yi: a vector of HRs with length = k

# sei: a vector of standard errors for each HR

# int.p: a vector of interaction p values with a flexible length < k

# at.int.p: position of each interaction p-values

# xlim: xlim for the forest plot

# at: at values for the barplot

# refline: vertical reference line value, normally indicating the null value of the plotted estimates (default==1.0)

# slab: a vector of rownames for the forest plot with length=n

# ilab: a matrix of data to be included on the left of the barplots and the p-value on the right of the barplots, with

# nrow=n, ncol=3 (vaccine #, placebo # and p-value)

# ilab.xpos: position of ilab

# ############################################

forestPlot<-function (yi,sei,int.p,at.int.p, xlim,at, refline = 1, slab,ilab, ilab.xpos,efac = 1, pch = 16, col

="darkgray", ...)

{

#setup

k <- length(yi)

rows <- k:1

ci.lb <- exp(log(yi)- 1.96 * sei)

ci.ub <- exp(log(yi )+ 1.96 * sei)

alim <- range(at)

# adding 4 or an arbitrary number of rows for headings and extra empty rows etc.

ylim <- c(-1.5, k + 4)

# start the plot

plot (NA, NA, xlim = xlim, ylim = ylim, xlab = "", ylab = "", yaxt = "n", xaxt = "n", xaxs = "i", bty = "n", ...)

abline(h = ylim[2] - 3, ...)

# user defined par

par.usr <- par("usr")

height <- par.usr[4] - par.usr[3]

lheight <- strheight("0")

cex <- 1

cex.lab <- 1

cex.axis <-1

# colors of the bars for significant and non-significant estimates, respectively.

col.bar <- ifelse(ci.lb>1|ci.ub<1,"red2","blue")

# add an axis at the bottom

axis(side = 1, at = at, labels = at, cex.axis = cex.axis,line=-1,...)

# forest plot part

for (i in 1:k) {

if (is.na(yi[i]))

next

segments(max(ci.lb[i], alim[1]), rows[i], min(ci.ub[i], alim[2]),lwd=2,rows[i],col=col.bar[i], ...)

if (ci.lb[i] >= alim[1]) {

segments(ci.lb[i], rows[i] - (height/150) * cex * efac, ci.lb[i], rows[i] + (height/150) * cex * efac,

lwd=2,col=col.bar[i],...)

}else {

polygon(x = c(alim[1], alim[1] + (1.4/100) * cex * (xlim[2] - xlim[1]), alim[1] + (1.4/100) * cex * (xlim[2] –

xlim[1]), alim[1]), y = c(rows[i], rows[i] + (height/150) * cex * efac, rows[i] - (height/150) * cex

* efac, rows[i]),border=col.bar[i],lwd=2,col = col.bar[i], ...)

}

if (ci.ub[i] <= alim[2]) {

segments(ci.ub[i], rows[i] - (height/150) * cex * efac, ci.ub[i], rows[i] + (height/150) * cex

*efac,lwd=2,col=col.bar[i], ...)

}else {

polygon(x = c(alim[2], alim[2] - (1.4/100) * cex * (xlim[2] - xlim[1]), alim[2] - (1.4/100) * cex *

(xlim[2] - xlim[1]), alim[2]), y = c(rows[i], rows[i] + (height/150) * cex * efac, rows[i] -

(height/150) * cex * efac, rows[i]),border=col.bar[i],lwd=2,col=col.bar[i], ...)

}

}

points(yi, rows, pch = pch, cex = cex*1.3,col=col.bar, ...)

# row names part(Group)

text(xlim[1], rows, slab, pos = 4, cex = cex, ...)

for (l in 1:NCOL(ilab)) {

text(ilab.xpos[l], rows, ilab[, l], pos =2,

cex = cex, ...)

}

segments(refline, ylim[1], refline, ylim[2] - 2, lty = "dotted", ...)

# HR and CI part

annotext <- format(round(cbind(yi,ci.lb,ci.ub),2),nsmall=2)

annotext <- cbind(annotext[, 1], " [", annotext[, 2], ", ", annotext[, 3], "]")

annotext <- apply(annotext, 1, paste, collapse = "")

text(x =0.5, rows, labels = annotext, pos = 2, cex = cex, ...)

#interatcion p

col.p<-rainbow(2,alpha = 0.2)

text(x = xlim[2], at.int.p, labels =int.p, pos = 2, cex = cex, ...)

#highlight significant interatcion p value

int.p<-int.p[int.p!="NA"]

N<-length(int.p)

if(N>0){

for(i in 1: N){

if(int.p[i]<=0.05){

rect(xleft=xlim[1],ybottom=at.int.p[i]- 0.9,

xright=xlim[2],ytop=at.int.p[i]+0.9,col=col.p[2],border=FALSE,density=NA)

}

}

}

#table titles

text(ilab.xpos[1:2], length(yi)+4, c(expression(bold("Vaccine")), expression(bold("Placebo"))), pos=2, cex = cex)

text(ilab.xpos[1:2], length(yi)+3, c(expression(bold("Infected")), expression(bold("Infected"))), pos=2, cex = cex)

text(ilab.xpos[1:2]+0.07, length(yi)+2,

c(expression(bold("(Incidence)")),expression(bold("(Incidence)"))),pos=2,cex = cex)

text(0.5,length(yi)+2,expression(bold("HR [95% CI]")),pos=2,cex = cex)

text(xlim[2]+0.07,length(yi)+3,expression(bold("Interaction")),pos=2,cex = cex)

text(xlim[2],length(yi)+2,expression(bold("P-value")),pos=2,cex = cex)

text(ilab.xpos[3],length(yi)+2,expression(bold("P-value")),pos=2,cex = cex)

text(xlim[1],length(yi)+3,expression(bold("Group")),pos=4,cex = cex)

text(xlim[1],length(yi)+2,expression(bold("(# Studies)")),pos=4,cex = cex)

abline(h = 0, ...)

invisible()

}

**Methods: Sensitivity Analysis of the Impact of Early Unblinding on Hazard Ratio (HR Vaccine: Placebo) Estimates**

A simulation-based sensitivity analysis was conducted to assess the impact of potential post-unblinding bias on the estimate of the vaccine effect in Step and Phambili combined. Such potential post-unblinding bias could be due to differential dropout or differential ascertainment of HIV infection among vaccine and placebo recipients.

For uninfected Step and Phambili study participants who dropped out early after unblinding, we assumed that time to infection since dropout was exponentially distributed with rates λ_p_ and λ_v_ for placebo and vaccine recipients, respectively, and censored at the maximal Step or Phambili study follow-up time observed in the dataset. We assumed that λ_p_ equaled the incidence rate observed among placebo recipients before unblinding, and that λ_v_ = RR_pud_* λ_p_, where RR_pud_ denotes the relative risk (vaccine: placebo) among dropouts post unblinding. For study participants who did not dropout early or dropped out early before unblinding, we assumed equal HIV risk factors between vaccine and placebo recipients.

For each of a range of fixed values of RR_pud_, we simulated the post-unblinding follow-up for dropouts and merged the simulated data with the observed data from dropouts and non-dropouts. We then estimated the overall HR (vaccine: placebo) using the simulated dataset based on a best-fit (AIC-selected) multivariable Cox model adjusting for potential baseline confounding factors as included in the original meta-analysis. The 2-sided P-value resulted from testing the null hypothesis of HR=1.0 was obtained. This procedure was repeated 1000 times and we reported the RR_pud_ corresponding to a median P-value ≥ 0.05.

**Methods: Analysis of Bias from Early Stopping in HVTN505**

A simulation-based analysis was conducted to assess the potential bias in the estimation of the HR introduced in HVTN505 as a result of early stopping. Of note, this bias adjustment only applies to those infections used in the stopping rule: infections between weeks 28 and 104 that were diagnosed before April 22, 2013 (the date of study unblinding).

In order to estimate the bias, a large number of simplified trials (10,000) were simulated under a simplified set of stopping rules, meant to approximate the actual stopping rules used in HVTN505.

The simplifying assumptions for the simulated study design are as follows, which are consistent with the HVTN505 protocol-specified analysis plan:

1. 1250 people enroll into each treatment group
2. The number of infections can be modeled as a cumulative binomial process, ignoring enrollment times and distributions of follow up times.
3. An analysis based on Relative Risk (RR), using only the count of infections in each group at each analysis point, is sufficiently close to approximate the actual analyses. (including CIs for stopping rules)
4. The true' infection rate in the placebo group (group 1) is 0.03.
5. The standard deviation of log viral load (VL) is 0.84 in both groups. This is based on the assumptions made in the protocol.
6. The true difference between the mean VLs is 0.
7. We do not include the monitoring for potential harm after each HIV infection

The stopping rules as implemented here include three potential analyses per simulated trial:

1. First look for non-efficacy after 43 infections, of which 30 have VL data: Construct 95% confidence intervals for RR for infections; also 95% confidence interval (CI) for VL difference in the subset of infections with VL available. Stop if the lower limit of the 95% CI for RR is > 0.5, and the upper limit is > 1.
2. If the study continues beyond A, check again at 60 infections, assuming 48 with VL data. Same criteria.
3. If the study continues beyond B, check again at final analysis.

One note, if there are not 60 infections in the entire 2500 people, the second analysis is not triggered and the final analysis on all infections follows after the first analysis at 43. Based on these assumptions, the simulations were run 10,000 times for each of a number of plausible true RRs. Results of these simulations are summarized in eFigure 14.

The next step is to start from the estimated RR and work backwards to estimate what the true RR was. We do this by solving for the true RR that would most likely lead to our biased estimate. We find $\tilde{\theta}$, the solution to $\hat{\theta}=\hat{\theta}+\beta\left( \tilde{\theta} \right)$, here $\hat{\theta}$ is our observed estimate and $\beta\left( \tilde{\theta} \right)$is the bias as a function of θ.^1^

In our case, we observed a RR of 1.28. In our simulations, this corresponds to a bias-corrected RR of approximately 1.25. On the figure, the dotted lines represent this back-calculation; the horizontal line at an observed RR of 1.28. This intersects our simulated Bias function at a true RR of approximately 1.25. The interpretation of this is that if the true RR were 1.25 we would expect to observe a RR of 1.28, on average. Therefore, our bias-adjusted estimate of the RR between week 28 and week 104 in HVTN505 is 1.25. Note that the observed RR here is based on the week 28+ infection split (the primary endpoint infection split) at the time that the study was stopped, and does not adjust for exact follow-up times. As a result, it is slightly different than the HR rates that were reported by the study team (1.25) for the same data and any subsequent estimates based on more recent infection numbers.

**TABLES**

**Table A: Comparisons of Follow-up Time and Number of HIV-1 Infections between the Meta-analysis and Prior Publications of Individual Trials.**

| **Vaccine regimen** | **Study** | **Maximal follow-up time since enrollment (months)** | | **Total number of HIV-1 infections** | |
| --- | --- | --- | --- | --- | --- |
|  |  | Our meta-analysis | Prior publications | Our meta-analysis | Prior publications |
| MRKAd5 | Step^2, 3^ | 51 | 51 | 187 | 82, 187 |
|  | Phambili^4, 5^ | 43 | 43 | 100 | 62, 100 |
| DNA/rAd5 | HVTN505^6^ | 58 | < 27 | 116 | 72 |
|  | IAVI001^7^ | 72 | < 30 | 0 | 0 |
|  | RV172^8^ | 75 | NA | 14 | 5 |
|  | HVTN204^9^ | 37 | 12 | 12 | 10 |

**Table B. Participant Baseline Characteristics.** The three phase 2b efficacy studies are Step, Phambili and HVTN505; all six studies include IAVI001, RV172 and HVTN204 in addition to the phase 2b studies.

| **Baseline Characteristics** | **Men** | | | | **Women** | | | |
| --- | --- | --- | --- | --- | --- | --- | --- | --- |
|  | **Three phase 2b studies*** | | **All six studies** | | **Three phase 2b studies** | | **All six studies** | |
|  | **Vaccine (n=2387)** | **Placebo (n=2386)** | **Vaccine (n=2635)** | **Placebo (n=2590)** | **Vaccine (n=742)** | **Placebo (n=751)** | **Vaccine (n=943)** | **Placebo (n=924)** |
| **Age (years)** | 29 (18,50) | 29 (18,50) | 28 (18,50) | 28 (18,50) | 26 (18,45) | 26 (18,45) | 26 (18,50) | 26 (18,50) |
| **Race/ethnic origin** |  |  |  |  |  |  |  |  |
| White | 1307 (55%) | 1339 (56%) | 1343 (51%) | 1368 (53%) | 23 (3%) | 31 (4%) | 51 (5%) | 68 (7%) |
| Black | 518 (22%) | 501 (21%) | 728 (28%) | 671 (26%) | 526 (71%) | 533 (71%) | 690 (73%) | 663 (72%) |
| Hispanic | 197 (8%) | 177 (7%) | 197 (7%) | 177 (7%) | 131 (18%) | 133 (18%) | 131 (14%) | 133 (14%) |
| Asian | 28 (1%) | 40 (2%) | 29 (1%) | 40 (2%) | 1 (0%) | 0 (0%) | 3 (0%) | 0 (0%) |
| Other | 337 (14%) | 329 (14%) | 338 (13%) | 334 (13%) | 61 (8%) | 54 (7%) | 68 (7%) | 60 (6%) |
| **Site of enrollment** |  |  |  |  |  |  |  |  |
| North America/Australia | 1840 (77%) | 1846 (77%) | 1885 (72%) | 1888 (73%) | 276 (37%) | 290 (39%) | 321 (34%) | 338 (37%) |
| South Africa | 222 (9%) | 219 (9%) | 270 (10%) | 272 (11%) | 178 (24%) | 181 (24%) | 243 (26%) | 241 (26%) |
| South America/Caribbean | 325 (14%) | 321 (13%) | 341 (13%) | 336 (13%) | 288 (39%) | 280 (37%) | 302 (32%) | 295 (32%) |
| East Africa | 0 (0%) | 0 (0%) | 139 (5%) | 94 (4%) | 0 (0%) | 0 (0%) | 77 (8%) | 50 (5%) |
| **Circumcision status** |  |  |  |  |  |  |  |  |
| Circumcised | 1813 (76%) | 1809 (76%) | 1813 (69%) | 1809 (70%) | NA | NA | NA | NA |
| Uncircumcised | 556 (23%) | 549 (23%) | 556 (21%) | 549 (21%) | NA | NA | NA | NA |
| Unknown | 18 (1%) | 28 (1%) | 266 (10%) | 232 (9%) | NA | NA | NA | NA |
| **Ad5 serostatus** |  |  |  |  |  |  |  |  |
| Positive | 1683 (71%) | 1687 (71%) | 1725 (65%) | 1718 (66%) | 114 (15%) | 121 (16%) | 141 (15%) | 143 (15%) |
| Negative | 704 (29%) | 699 (29%) | 910 (35%) | 872 (34%) | 628 (85%) | 630 (84%) | 801 (85%) | 781 (85%) |
| **HSV-2 serostatus** |  |  |  |  |  |  |  |  |
| Positive | 300 (13%) | 318 (13%) | 300 (11%) | 318 (12%) | 90 (12%) | 86 (11%) | 90 (10%) | 86 (9%) |
| Negative | 829 (35%) | 819 (34%) | 829 (31%) | 819 (32%) | 87 (12%) | 95 (13%) | 87 (9%) | 95 (10%) |
| **Risk score** |  |  |  |  |  |  |  |  |
| Low (risk score=0) | 190 (8%) | 176 (7%) | 342 (13%) | 288 (11%) | 70 (9%) | 85 (11%) | 188 (20%) | 176 (19%) |
| Median (risk score=1 or 2) | 1112 (47%) | 1136 (48%) | 1238 (47%) | 1243 (48%) | 281 (38%) | 273 (36%) | 390 (41%) | 361 (39%) |
| High (risk score>2) | 1085 (45%) | 1074 (45%) | 1117 (42%) | 1089 (42%) | 391 (53%) | 393 (52%) | 419 (44%) | 399 (43%) |

Note: data are median (range) or number (%), unless otherwise indicated. *Includes 44 transgender women in HVTN505.

**Table C: Estimates of Time-dependent Ad5-dose Response Based on Step and Phambili, with and without including HVTN505 in the Ad5 Cohort.**

| **Number of Ad5** | **Ad5 cohort** | | | | | | | |
| --- | --- | --- | --- | --- | --- | --- | --- | --- |
|  | **Step + Phambili + HVTN505** | | | | **Step + Phambili** | | | |
|  | **HR** | **95% CI** | **P** | **Interaction P** | **HR** | **95% CI** | **P** | **Interaction P** |
| **1** | 0.91 | (0.63, 1.29) | 0.59 | **0.14** | 1.57 | (0.80, 3.07) | 0.19 | **0.79** |
| **2** | 1.52 | (1.02, 2.25) | 0.04 |  | 1.52 | (1.02, 2.26) | 0.04 |  |
| **3** | 1.30 | (0.93, 1.81) | 0.12 |  | 1.30 | (0.93, 1.82) | 0.12 |  |

**Table D. Sensitivity Analysis Example Scenario Based on Step and Phambili.** Hypothetical person-years (pys) were calculated for those dropouts that occurred after unblinding assuming those participants had stayed in the study until the maximal length of either Step or Phambili. Given the observed total pys and number of infections, a 10:21 (V: P) split of 31 additional infections is one possible scenario that would reverse the observed significant vaccine-increased risk. This was based on a two-sample Poisson test using a 1-sided type I error rate of 0.05.

|  | **Hypothetical scenario** | | | **Observed data** | | |
| --- | --- | --- | --- | --- | --- | --- |
| **Group** | **Pys**  **Post Dropout**  **Unblinded** | **# of Infections post-dropout unblinded** | **Annual Incidence** | **Total Pyrs** | **Total # of Infections** | **Annual Incidence before unblinding** |
| Vaccine | 550 | 10 | 1.8% | 5379 | 169 | 3.1% |
| Placebo | 607 | 21 | 3.5% | 5434 | 118 | 2.3% |

**Figure A. Kaplan-Meier Curves and Their Difference of Cumulative Incidence of HIV-1 Infection for the Individual Studies: Step, Phambili, HVTN505 and the Triad Studies in the MITT Cohort.** The MITT cohort included all randomly assigned participants HIV uninfected at study entry, who received at least one dose of vaccine or placebo. P-values were based on a 2-sided Wald test of whether the HR (V: P) differed from 1.0 as estimated from the best-fit multivariable Cox model.

**Figure B. Kaplan-Meier Curves and Their Difference of Cumulative Incidence of HIV-1 Infection for the Individual Studies: Step, Phambili, HVTN505 and the Triad Studies in the Ad5 Cohort.** The Ad5 cohort included MITT participants who received at least one dose of rAd5-HIV vaccine or rAd5-placebo. P-values were based on a 2-sided Wald test of whether the HR (V: P) differed from 1.0 as estimated from the best-fit multivariable Cox model.

**Figure C. Estimated HRs in the MITT Cohort (Panel A) and the Ad5 Cohort (Panel B) Based on All Six Studies.** The MITT cohort included all randomly assigned participants HIV uninfected at study entry, who received at least one dose of vaccine or placebo; the Ad5 cohort included MITT participants who received at least one dose of rAd5-HIV vaccine or rAd5-placebo. Red lines are 95% confidence intervals indicating HRs significantly different from 1.0; blue lines are 95% confidence intervals indicating HRs not significantly different from 1.0.

**Figure D. Power Calculations for HR Comparisons between HVTN505 vs. Step and Phambili Combined Based on Sample Sizes in the MITT Cohort (Panel A) and the Ad5 cohort (Panel B).** The X- and Y-axes indicate the assumed true HRs of Step+Phambili and HVTN505, respectively; percentages on the other two axes indicate the statistical power in comparing each pair of HRs. The MITT cohort included all randomly assigned participants HIV uninfected at study entry, who received at least one dose of vaccine or placebo; the Ad5 cohort included MITT participants who received at least one dose of rAd5-HIV vaccine or rAd5-placebo. Indicated by the numbers next to each slanted line is the power to detect various HR differences between a give true HR from HVTN505 and a given true HR from Step+Phambili combined. In particular, power equals 56.8% and 76.3% when HRs equal their point estimates observed in the MITT cohort and Ad5 cohort, respectively.

**Figure E. Nonparametric Instantaneous HR of Infection in the MITT Cohort (Panel A) and the Ad5 Cohort (Panel B) Based on Step, Phambili and HVTN505.** The MITT cohort included all randomly assigned participants HIV uninfected at study entry, who received at least one dose of vaccine or placebo; the Ad5 cohort included MITT participants who received at least one dose of rAd5-HIV vaccine or rAd5-placebo. The test for a time-varying HR gave P = 0.84 (MITT cohort) and P= 0.50 (Ad5 cohort).

**Figure F. Sensitivity Analysis to Assess the Impact of Post-unblinding Bias in Step and Phambili Combined.** For each hypothetical value of RR (V: P) post-unblinding among dropouts, the probability of detecting an overall increased risk of HIV infection (black curve with scales on the left axis), and the corresponding overall HR (red dotted curve with scales on the right axis) were calculated based on 1000 simulated datasets. This figure indicates that, if there were truly no vaccine-increased risk, then the vaccine recipients who terminated early would need to have 45% lower risk of HIV-1 infection post-unblinding compared to the placebo recipients who terminated early, in order to achieve the observed HR estimate of 1.41 in Step and Phambili combined.

**Figure G. Estimated HRs by Study and Follow-up Time Period.** Red lines are 95% confidence intervals indicating HRs significantly different from 1.0; blue lines are 95% confidence intervals indicating HRs not significantly different from 1.0.

**Figure H. Estimated HRs in Subgroups Using Follow-up Time Overall (Panel A), before 18 Months (Panel B) and after 18 Months (Panel C) Based on Step and Phambili.** Red lines are 95% confidence intervals indicating HRs significantly different from 1.0; blue lines are 95% confidence intervals indicating HRs not significantly different from 1.0. Shaded rows indicate significantly different HRs within each pair of subgroups.

**Figure I. Estimated HRs in the MITT Cohort (Panel A) and Ad5 Cohort (Panel B) among Circumcised Ad5-negative Men in Step, Phambili and HVTN505.** The MITT cohort included all randomly assigned participants HIV uninfected at study entry, who received at least one dose of vaccine or placebo; the Ad5 cohort included MITT participants who received at least one dose of rAd5-HIV vaccine or rAd5-placebo. Red lines are 95% confidence intervals indicating HRs significantly different from 1.0; blue lines are 95% confidence intervals indicating HRs not significantly different from 1.0.

**Figure J: Estimated HRs by Follow-up Time Period in Subgroups Defined by Baseline Circumcision Status and Ad5 Serostatus Based on Step and Phambili.** Red lines are 95% confidence intervals indicating HRs significantly different from 1.0; blue lines are 95% confidence intervals indicating HRs not significantly different from 1.0. Shaded rows indicate significantly different HRs within each pair of subgroups.

**Figure K: Summary of Estimated HRs and Interaction Test P-values by Follow-up Time Period and Baseline Subgroup Based on Step and Phambili Combined.** The P and Q above the horizontal lines are 2-sided p-values and q-values for testing that the HRs differ in the early vs. late follow-up periods, for the group indicated in the row. The P and Q beside the vertical lines are 2-sided p-values and q-values for testing that the HRs differ between the two subgroups, for the follow-up period indicated in the column.

**Figure L. Bias-corrected Relative Risk to Assess the Impact of Early Stopping in HVTN505.** Each dot corresponds to one set of 10,000 simulations, where the x-value gives the assumed true RR for the simulations, and the y-value gives the geometric mean RR across the 10,000 simulations. The solid line is the identity line, for comparison; the dotted horizontal and vertical segments are displayed to clarify where the observed HVTN505 RR (as of April 22, 2013) falls on the graph and what our best guess of the corresponding unbiased RR would be from these results.

**REFERENCES**

1. Proschan M, Lan K, Wittes J. *Statistical Monitoring of Clinical Trials: A Unified Approach.* New York, NY: Springer; 2006.

2. Buchbinder SP, Mehrotra DV, Duerr A, et al. Efficacy assessment of a cell-mediated immunity HIV-1 vaccine (the Step Study): A double-blind, randomised, placebo-controlled, test-of-concept trial. *Lancet.* Nov 29 2008; 372(9653):1881-1893.

3. Duerr A, Huang Y, Buchbinder S, et al. Extended follow-up confirms early vaccine-enhanced risk of HIV acquisition and demonstrates waning effect over time among participants in a randomized trial of recombinant adenovirus HIV vaccine (Step Study). *J Infect Dis.* Jul 15 2012;206(2):258-266.

4. Gray GE, Allen M, Moodie Z, et al. Safety and efficacy of the HVTN 503/Phambili study of a clade-B-based HIV-1 vaccine in South Africa: A double-blind, randomised, placebo-controlled test-of-concept phase 2b study. *Lancet Infect Dis.* Jul 2011;11(7):507-515.

5. Gray GE, Moodie Z, Metch B, Gilbert PB, Bekker LG, et al. (2014) Recombinant adenovirus type 5 HIV gag/pol/nef vaccine in South Africa: unblinded, long-term follow-up of the phase 2b HVTN 503/Phambili study. Lancet Infect Dis 14: 388-396.

6. Hammer SM, Sobieszczyk ME, Janes H, et al. Efficacy trial of a DNA/rAd5 HIV-1 preventive vaccine. *N Engl J Med.* Nov 28 2013;369(22):2083-2092.

7. Jaoko W, Karita E, Kayitenkore K, et al. Safety and immunogenicity study of multiclade HIV-1 adenoviral vector vaccine alone or as boost following a multiclade HIV-1 DNA vaccine in Africa. *PLoS One.* 2010;5(9):e12873.

8. Kibuuka H, Kimutai R, Maboko L, et al. A phase 1/2 study of a multiclade HIV-1 DNA plasmid prime and recombinant adenovirus serotype 5 boost vaccine in HIV-uninfected East Africans (RV 172). *J Infect Dis.* Feb 15 2010;201(4):600-607.

9. Churchyard GJ, Morgan C, Adams E, et al. A phase IIA randomized clinical trial of a multiclade HIV-1 DNA prime followed by a multiclade rAd5 HIV-1 vaccine boost in healthy adults (HVTN204). *PLoS One.* 2011;6(8):e21225.
